# Association between metabolic phenotype and urinary albumin‐creatinine ratio in Chinese community adults: A cross‐sectional study

**DOI:** 10.1111/1753-0407.13302

**Published:** 2022-08-30

**Authors:** Yue Zhang, Binqi Li, Yang Liu, Wenxing Gao, Kang Chen, Anping Wang, Xulei Tang, Li Yan, Zuojie Luo, Guijun Qin, Lulu Chen, Qin Wan, Zhengnan Gao, Weiqing Wang, Guang Ning, Yiming Mu

**Affiliations:** ^1^ Department of Endocrinology The First Clinical Medical Center of Chinese People's Liberation Army General Hospital Beijing China; ^2^ Medical School of Chinese PLA Beijing China; ^3^ School of Medicine Nankai University Tianjin China; ^4^ The First Hospital of Lanzhou University Lanzhou Gansu China; ^5^ Sun Yat‐sen Memorial Hospital Sun Yat‐sen University Guangzhou China; ^6^ The First Affiliated Hospital of Guangxi Medical University Nanning China; ^7^ The First Affiliated Hospital of Zhengzhou University Zhengzhou China; ^8^ Union Hospital Tongji Medical College Wuhan China; ^9^ Affiliated Hospital of Luzhou Medical College Luzhou China; ^10^ Dalian Municipal Central Hospital Dalian China; ^11^ Ruijin Hospital Shanghai Jiao Tong University School of Medicine Shanghai China

**Keywords:** chronic kidney disease, metabolic phenotype, obesity, urinary albumin‐creatinine ratio, 慢性肾病, 代谢表型, 肥胖, 尿白蛋白/肌酐

## Abstract

**Background:**

Urinary albumin‐creatinine ratio (UACR) is a sensitive marker of kidney injury. This study analyzed the prevalence of different metabolic phenotypes and investigated their relationship with UACR in Chinese community adults.

**Methods:**

This study involved 33 303 participants over 40 years old from seven centers across China. They were stratified into six groups according to their body mass index (BMI) and metabolic status: metabolically healthy normal weight (MHNW), metabolically healthy overweight (MHOW), metabolically healthy obesity (MHO), metabolically unhealthy normal weight (MUNW), metabolically unhealthy overweight (MUOW), and metabolically unhealthy obesity (MUO). Increased albuminuria was defined as a UACR ≥30 mg/g.

**Results:**

The percentages of MHNW, MHOW, MHO, MUNW, MUOW, and MUO were 27.6%, 15.9%, 4.1%, 19.8%, 22.5%, and 9.6%, respectively. Multiple logistic regression analysis showed that the MHO group (odds ratio [OR] 1.205; 95% CI, 1.081‐1.343), MUNW group (OR 1.232; 95% CI, 1.021‐1.486), MUOW group (OR 1.447; 95% CI, 1.303‐1.607), and MUO group (OR 1.912; 95% CI, 1.680‐2.176) were at higher risk of increased albuminuria compared to the MHNW group. Subgroup analysis indicated that the risk of increased albuminuria was further elevated among regular smokers in men aged 40 to 55 years old with abdominal obesity.

**Conclusions:**

Among Chinese community adults, increased albuminuria was associated with increased BMI whether metabolism was normal or not, and those with abnormal metabolism were at greater risk of increased albuminuria than those with normal metabolism. These findings suggest that overweight or obesity or metabolic abnormalities are risk factors for chronic kidney disease.

## Graphical abstract

Highlights

This is the first multicenter, large‐sampled cross‐sectional study of the association between different metabolic phenotypes and urinary albumin‐creatinine ratio (UACR) in Chinese community adults. Our study may be helpful for people with increased albuminuria. We recommend that they have their UACR checked regularly and maintain a healthy lifestyle to reduce their risk of chronic kidney disease.

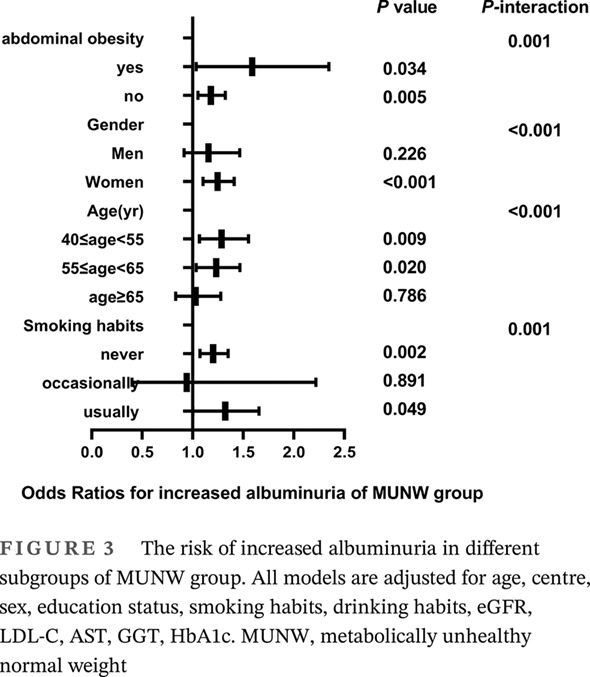

## 摘要

**背景:**

尿白蛋白/肌酐比值(UACR)是反映肾脏损伤的有效指标。本研究分析了不同代谢表型在中国社区成人中的患病率, 并探讨了它们与UACR的关系。

**方法:**

这项研究纳入来自中国7个中心的33303名40岁以上的参与者。根据体重指数(BMI)和代谢状态将受试者分为6组:代谢健康正常体重(MHNW), 代谢健康超重(MHOW), 代谢健康肥胖(MHO), 代谢不健康正常体重(MUNW), 代谢不健康超重(MUOW)和代谢不健康肥胖(MUO)。UACR≥ 30 mg/g被定义为蛋白尿增加。

**结果:**

MHNW, MHOW, MHO, MUNW, MUOW和MUO所占比例分别为27.6%, 15.9%, 4.1%, 19.8%, 22.5%和9.6%。多因素Logistic回归分析显示, MHO组(OR=1.205[95%CI:1.081~1.343]), MUNW组(OR=1.232[95%CI:1.021~1.486]), MUOW组(OR=1.447[95%CI:1.303~1.607])和MUO组(OR=1.912[95%CI:1.680~2.176])发生蛋白尿增加的风险高于MHNW组。亚组分析表明, 在40至55岁腹部肥胖的男性中, 经常吸烟的人蛋白尿增加的风险进一步增加。

**结论:**

在中国社区成年人中, 无论代谢是否正常, 尿白蛋白增加都与BMI增加相关, 代谢异常的人比代谢正常的人更容易出现蛋白尿增加的危险。这些发现表明, 超重, 肥胖和代谢异常是慢性肾脏疾病的风险因素。

## 1 INTRODUCTION

Chronic kidney disease (CKD) is a global public health challenge. It will eventually develop into uremia with the progress of proteinuria, with a poor prognosis and a heavy economic burden of relying solely on replacement therapy to extend life.^1^ In 2017, the global prevalence of CKD was estimated at 9.1%, ranking as the 12th leading cause of death.^2^ CKD has an insidious onset, and therefore early diagnosis and treatment are critical to prevent renal insufficiency and delay the progression of uremia. The urinary albumin‐creatinine ratio (UACR) is currently used in clinical screening^3^ and has the advantage of being convenient and accurate. UACR is a sensitive marker of early renal injury and an accurate predictor of cardiovascular events.^4^ Therefore, routine screening of UACR is necessary to identify high‐risk individuals in clinical practice.

With rapid economic development and lifestyle changes, the prevalence of overweight and obesity has increased significantly worldwide. Given this growing trend, it is expected that up to 57.8% of the population will be overweight or obese by 2030.^5^ The results of a large European population survey showed that obesity is one of the strongest risk factors for the new onset of CKD.^6^ Metabolic health is also receiving increasing attention. Metabolic health refers to the absence of any of the following: hyperglycemia, hypertension, dyslipidemia, high high‐density lipoprotein cholesterol (HDL‐C) level, or abdominal obesity.^7^ Metabolic abnormalities are associated with CKD, and the risk of developing CKD increases with the number of metabolically abnormal components.^8^ However, not all obese subjects develop metabolic abnormalities. The prognostic value of the metabolically healthy obesity (MHO) group is a controversial topic; some studies report that MHO is associated with lower mortality^9^ but with a higher risk of developing CKD^10^. It is necessary to note that many cohort studies have shown that metabolic phenotypes change over time,^10^ so it is important to determine whether each phenotype contributes to health and leads people to change to a beneficial phenotype.

To our knowledge, there are very few studies on the association of increased proteinuria with different metabolic phenotypes. Therefore, the purpose of this study was to explore the prevalence of different metabolic types, to determine whether there are differences in the increase of albuminuria in different metabolic phenotypes, and to provide recommendations for the early prevention of CKD.

## 2 METHODS

### 2.1 Participants and study design

**FIGURE 1:**
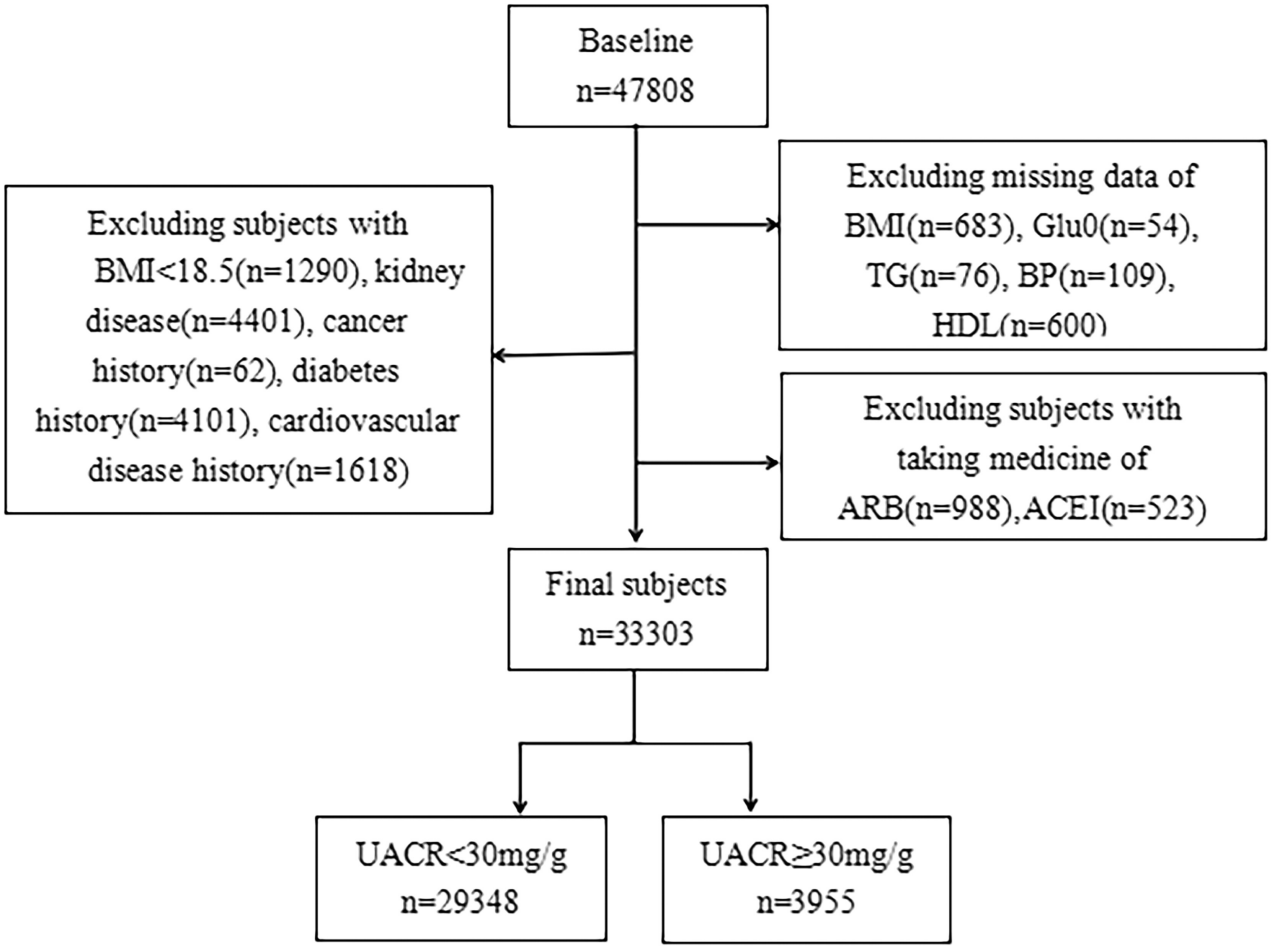
Flow chart of study population

Study participants were from the REACTION (Risk Evaluation of Cancers in Chinese Diabetic Individuals study),^11^ which recruited 47 808 participants over 40 years old in 2012 from seven geographically dispersed regional centers in China (Dalian, Guangzhou, Lanzhou, Luzhou, Shanghai, Zhengzhou, and Wuhan). Participants who were diagnosed with primary kidney disease, those with cancer history, cardiovascular disease (CVD) history, diabetes history, a body mass index (BMI) < 18.5, those who previously used lipid‐lowering drugs, angiotensin‐converting enzyme inhibitor (ACEI) drugs, or angiotensin receptor blocker (ARB) drugs, or those with missing important data were excluded from this study. Finally, 33 303 participants were included (Figure 1). This study was conducted according to the Declaration of Helsinki, and the protocol was approved by the Clinical Research Ethics Committee of Rui‐Jin Hospital affiliated with the School of Medicine, Shanghai Jiao Tong University. Informed consent was obtained from all participants before the study.

### 2.2 Data collection

Basic information about the participants was obtained by trained investigators using a standardized questionnaire. This included demographic characteristics (gender, age), history of underlying diseases (diabetes, hypertension, CVD), medication history (hypoglycemic drugs, antihypertensive drugs, etc.), and lifestyle habits such as smoking (never smoked, occasional smoking [less than one cigarette per day or less than seven cigarettes per week], and regular smoking [at least one cigarette per day]) and drinking (never drank, occasional drinking [less than once a week], and regular drinking [at least once a week for more than 6 months]. Standardized measurements of height, weight, waist circumference (WC), and blood pressure were taken. Participants were asked to remove shoes, hats, and clothing and to rest in a seated position for 5 min before blood pressure measurement, and three measurements were taken using a mercury sphygmomanometer and averaged. WC was defined as the circumference of the abdomen at the midpoint of the line connecting the lower edge of the rib cage and the iliac crest.

### 2.3 Biochemical evaluation

Participants underwent an oral glucose tolerance test after fasting (≥10 h) followed by early morning fasting blood sample collection. Morning urine samples were collected to determine urinary albumin concentration and creatinine by chemiluminescence immunoassay. UACR concentration was calculated as urinary albumin (mg)/urinary creatinine (g). The coefficient of variation is the ratio of SD to mean. The biochemical indicators tested included aspartate aminotransferase (AST), alanine aminotransferase (ALT), serum creatinine (SCr), glycosylated hemoglobin (HbA1c), glutamyl transferase (GGT), fasting blood glucose (FBG), 2‐hour postprandial blood glucose (PBG), rapid insulin assay (0 min, 120 min), and low‐density lipoprotein cholesterol (LDL‐C), HDL‐C, total cholesterol (TC), and triglycerides (TG). Estimated glomerular filtration rate (eGFR) was estimated from a simplified equation developed from data from the Modification of Diet in Renal Disease (MDRD) study^12^ as follows: eGFR (ml/min/1.73 m^2^) = 186 × [SCr(mg/dl)/88.4]^−1.154^ × (age)^−0.203^ × (0.742 if female) × 1.233; insulin resistance (HOMA‐IR [homeostatic model assessment of insulin resistance]) was assessed using a steady‐state model calculated as fasting plasma insulin (U/L) × FBG (mmol/L)/22.5.

### 2.4 Definition of variables

BMI was defined by weight (kg) divided by height squared (m) and was classified as underweight (<18.5 kg/m^2^), normal weight (18.5 ≤ BMI < 24), overweight (24 ≤ BMI < 28), and obese (BMI ≥ 28) according to the criteria of the Working Group on Obesity in China (WGOC).^13^ Underweight participants were removed from the analysis. Metabolically healthy was defined as free of metabolic syndrome (MS) components according to the National Cholesterol Education Program Adult Treatment Panel III criteria,^14^ and WC was not used as a criterion due to covariance with BMI. We assessed abdominal obesity based on waist‐hip ratio (WHR). Abdominal obesity is defined^15^ as WHR > 0.9 for men and WHR > 0.85 cm for women. Participants were stratified into metabolically healthy normal weight (MHNW), metabolically healthy overweight (MHOW), MHO, metabolically unhealthy normal weight (MUNW), metabolically unhealthy overweight (MUOW), and metabolically unhealthy obesity (MUO) groups according to BMI and metabolic status (Table 1). Increased albuminuria was defined as UACR ≥ 30 mg/g according to the Kidney Disease: Improving Global Outcomes (KDIGO) CKD guidelines, suggesting renal damage. UACR was divided into the following two groups: normal albuminuria (UACR < 30 mg/g) and increased albuminuria (UACR ≥ 30 mg/g).

**TABLE 1:** Criteria of metabolic phenotype

BMI	Metabolic risk factors	Metabolic phenotype
18.5 ≤ BMI < 24	0	MHNW
24 ≤ BMI < 28	0	MHOW
BMI ≥ 28	0	MHO
18.5 ≤ BMI < 24	≥1	MUNW
24 ≤ BMI < 28	≥1	MUOW
BMI ≥ 28	≥1	MUO

*Notes*: Metabolic risk factors include (1) hyperglycemia (FPG ≥110 ml/dL or use of hypoglycemic drugs), (2) hypertension (SBP ≥130 mmHg or DBP ≥85 mmHg or use of antihypertensive drugs), (3) dyslipidemia (TG ≥150 mg/dl or use of lipid‐lowering drugs), and (4) HDL‐C level ≥40 mg/dl (males) or ≥50 mg/dl (females).

Abbreviations: BMI, body mass index; DBP, diastolic blood pressure; FPG, fasting plasma glucose; HDL‐C, high‐density lipoprotein cholesterol; MHNW, metabolically healthy normal weight; MHO, metabolically healthy obesity; MHOW, metabolically healthy overweight; MUNW, metabolically unhealthy normal weight; MUO, metabolically unhealthy obesity; MUOW, metabolically unhealthy overweight; SBP, systolic blood pressure; TG, triglycerides.

### 2.5 Statistical methods

Categorical variables were expressed as percentages (%), continuous variables were expressed as mean ± SD or median (interquartile range, IQR), and the Kolmogorov‐Smirnov test was used to determine whether the variables were normally distributed. Differences between groups of continuous variables were analyzed by one‐way analysis of variance (ANOVA) or Kruskal‐Wallis test, and comparisons of categorical variables were performed by the chi‐square test. Pearson correlation analysis was used for normal data, and the Spearman rank test was used to assess the correlation for nonparametric data. Logistic regression analysis was used to estimate odds ratio (OR) and 95% CI to explore the association of different phenotypes with increased albuminuria. Model 1 was unadjusted. Model 2 was adjusted for age, center, and sex. Model 3 was further adjusted for educational status, smoking habits, and alcohol consumption habits. Model 4 was further adjusted for eGFR, LDL‐C, AST, GGT, and HbA1c. Stratified analysis was performed with age (40 ≤ age < 55 years, 55 ≤ age < 65 years, age ≥ 65 years), sex (male, female), abdominal obesity (yes, no), and smoking habits (never, occasionally, and regularly) to further examine the association between different metabolic phenotypes and increased albuminuria; potential multiple confounders were adjusted. The software used for data analysis was SPSS Version 25.0 (IBM). Results were considered statistically significant if the two‐sided *p*‐values were <0.05.

## 3 RESULTS

### 3.1 Clinical characteristics of participants

Ultimately, we included 33 303 participants (9554 men and 23 749 women). Table 2 shows the clinical and biochemical characteristics of the six groups of participants. The proportions of MHNW, MHOW, MHO, MUNW, MUOW, and MUO participants were 27.6% (7218/1995), 15.9% (4096/1286), 4.1% (1087/295), 19.8% (4449/2152), 22.5% (4743/2764), and 19.6% (2156/1062). Among men, the MUOW population had the largest share and the MHO population the smallest. Among women, the MHNW population had the largest share and the MHO population the smallest. Metabolic indicators such as FBG, PBG, LDL, TG, systolic blood pressure (SBP), diastolic blood pressure (DBP), HbA1c, and HOMA‐IR were increased in the other groups compared to the MHNW group. Among all participants, the median of UACR was 9.43, and the coefficient of variation was 14.069%. There was a significant difference in the prevalence of increased albuminuria between these six groups, with only 9.6% of subjects in the MHNW group having increased albuminuria compared to 11.4% in the MHO group, 12.6% in the MUNW group, 14.1% in the MUOW group, and 16.9% in the MUO, which was twice as high as in the MHNW group.

**TABLE 2:** Characteristics of study population by metabolic phenotype

	Metabolically healthy			Metabolically unhealthy			
Variables	Normal weight (*n* = 9213)	Overweight (*n* = 5328)	Obesity (*n* = 1382)	Normal weight (*n* = 6601)	Overweight (*n* = 7507)	Obesity (*n* = 3218)	*P* value
Women, *n* (%)	7218 (78.3%)	4096 (76.1%)	1087 (78.7%)	4449 (67.4%)	4743 (63.2%)	2156 (67.0%)	<0.001
Men, *n* (%)	1995 (21.7%)	1286 (23.9%)	295 (21.3%)	2152 (32.6%)	2764 (36.8%)	1062 (33.0%)	<0.001
Age, years	54.9 (8.41)	55.2 (10.8)	56.1 (12.2)	58.1 (11.8)	58.3 (10.5)	59.1 (11.3)	<0.001
BMI, kg/m^2^	21.7 (1.4)	25.5 (1.8)	29.5 (2.5)	22.3 (2.1)	25.8 (1.9)	29.6 (2.5)	<0.001
eGFR, ml/min/1.73 m^2^	117.9 (25.3)	120.7 (26.2)	121.8 (27.8)	112.5 (24.7)	111.8 (24.4)	113.1 (26.0)	<0.001
FBG, mmol/L	5.2 (0.6)	5.4 (0.6)	5.5 (0.7)	5.6 (0.9)	5.7 (1.1)	5.9 (1.2)	<0.001
PBG, mmol/L	6.4 (2.1)	7.2 (2.4)	7.3 (2.7)	7.3 (3.1)	7.8 (3.6)	8.3 (4.1)	<0.001
HDL‐C, mmol/L	1.3 (0.4)	1.2 (0.4)	1.1 (0.3)	1.4 (0.4)	1.3 (0.4)	1.3 (0.4)	<0.001
LDL‐C, mmol/L	2.7 (0.9)	2.8 (1.2)	2.8 (1.1)	3.0 (1.2)	3.1 (1.2)	3.2 (1.1)	<0.001
TC, mmol/L	4.7 (1.1)	4.7 (1.1)	4.6 (1.4)	5.3 (1.4)	5.3 (1.2)	5.3 (1.4)	<0.001
TG, mmol/L	1.1 (0.7)	1.3 (0.6)	1.3 (0.6)	1.5 (1.1)	1.8 (1.2)	1.9 (1.2)	<0.001
SBP, mmHg	116 (13)	120.7 (14.3)	124 (16.3)	136.3 (20.0)	139.6 (20.3)	142.6 (22.7)	<0.001
DBP, mmHg	70.7 (8.1)	73.7 (11)	76.0 (11.7)	79.6 (13.3)	82.0 (13.7)	83.6 (13.0)	<0.001
HbA1c, %	5.7 (0.5)	5.8 (0.6)	5.9 (0.6)	5.9 (0.6)	6.0 (0.7)	6.1 (0.7)	<0.001
SCr, mg/dl	64.7 (12.0)	64.7 (12.6)	64.2 (13)	66.2 (13.2)	67.1 (13.7)	66.8 (15.7)	<0.001
WC, cm	78.0 (10.0)	87.0 (9.0)	96.0 (10)	81.0 (9.7)	90.0 (9.0)	98.0 (10.0)	<0.001
WHR, %	0.85 (0.09)	0.88 (0.08)	0.90 (0.08)	0.89 (0.09)	0.91 (0.08)	0.92 (0.07)	<0.001
HOMA‐IR	1.4 (0.8)	1.8 (1.1)	2.4 (1.5)	1.8 (1.1)	2.2 (1.4)	2.9 (2.0)	<0.001
AST, U/L	19.0 (7.0)	19.0 (7.0)	19.0 (8.0)	21.0 (7.0)	21.0 (8.0)	22.0 (10.0)	<0.001
ALT, U/L	13.0 (8.0)	14 (10.0)	16.0 (12.0)	15.0 (9.0)	17.0 (11.0)	19.0 (14.0)	<0.001
GGT, U/L	16.0 (10.0)	18.0 (13.0)	20.0 (15.0)	21.0 (15.0)	25.0 (21.0)	28.0 (24.0)	<0.001
UACR	9.0 (11.0)	9.1 (10.9)	9.2 (12.4)	9.8 (13.6)	9.9 (13.9)	10.6 (15.9)	<0.001

*Note*: Data were medians (IQR) for skewed variables or numbers (proportions) for categorical variables.

Abbreviations: ALT, alanine aminotransferase; AST, aspartate aminotransferase; BMI, body mass index; DBP, diastolic blood pressure; eGFR, estimated glomerular filtration rate; FBG, fasting blood glucose; GGT, glutamyl transferase; HbA1c, glycosylated hemoglobin; HDL‐C, high‐density lipoprotein cholesterol; HOMA‐IR, homeostatic model assessment of insulin resistance; IQR, interquartile range; LDL‐C, low‐density lipoprotein cholesterol; PBG, 2‐hour postprandial blood glucose; SBP, systolic blood pressure; SCr, serum creatinine; TC, total cholesterol; TG, triglycerides; UACR, urinary albumin‐creatinine ratio; WC, waist circumference; WHR, waist‐hip ratio.

### 3.2 Logistic regression analysis of the correlation between different metabolic phenotypes and elevated UACR

The OR of increased albuminuria in the remaining groups were estimated using the MHNW group as the reference group (Table 3), and the OR of all groups except the MHOW group were statistically significant in all four models and were greater than 1, suggesting an elevated risk of increased albuminuria. In model 4, after adjusting for possible potential confounders, the risk of occurrence of increased albuminuria was also elevated in the MHO group (OR 1.205; 95% CI, 1.081‐1.343; *P* = .029) compared with the MHNW group in the metabolically normal population, suggesting that MHO is not a benign state. In the population with metabolic abnormalities, the OR for the occurrence of elevated UACR increased with increasing BMI (MUNW group [OR 1.232; 95% CI, 1.021–1.486; *p* < 0.001], MUOW group [OR 1.447; 95% CI, 1.303–1.607; *p* < 0.001], MUO group [OR 1.912; 95% CI, 1.680–2.176; *p* < 0.001]).

**TABLE 3:** Risk of elevated albuminuria in relation to different metabolic phenotypes among all participants

	Model 1 OR (95% CI)	Model 2 OR (95% CI)	Model 3 OR (95% CI)	Model 4 OR (95% CI)
MHNW	Reference	Reference	Reference	Reference
MHOW	0.920 (0.819–1.034) *p* = 0.164	0.937 (0.831–1.056) *p* = 0.284	0.930 (0.824–1.048) *p* = 0.234	0.913 (0.809–1.030) *p* = 0.138
MHO	1.208 (1.009–1.446) *p* = 0.040*	1.292 (1.162–1.436) *p* = 0.005**	1.284 (1.067–1.422) *p* = 0.008**	1.205 (1.081–1.343) *p* = 0.029*
MUNW	1.363 (1.232–1.506) *p* < 0.001***	1.305 (1.083–1.572) *p* < 0.001***	1.289 (1.155–1.553) *p* < 0.001***	1.232 (1.021–1.486) *p* = 0.001**
MUOW	1.544 (1.404–1.698) *p* < 0.001***	1.612 (1.457–1.783) *p* < 0.001***	1.596 (1.442–1.766) *p* < 0.001***	1.447 (1.303–1.607) *p* < 0.001***
MUO	1.917 (1.708–2.151) *p* < 0.001***	2.254 (1.991–2.552) *p* < 0.001***	2.204 (1.945–2.497) *p* < 0.001***	1.912 (1.680–2.176) *p* < 0.001***

*Notes*: Model 1‐unadjusted; model 2‐adjusted for age, center, sex; model 3‐further adjusted for education status, smoking habits, drinking habits; model 4‐further adjusted for eGFR, LDL‐C, AST, GGT, HbA1c.

Abbreviations: AST, aspartate aminotransferase; eGFR, estimated glomerular filtration rate; GGT, glutamyl transferase; HbA1c, glycosylated hemoglobin; LDL‐C, low‐density lipoprotein cholesterol; MHNW, metabolically healthy normal weight; MHO, metabolically healthy obesity; MHOW, metabolically healthy overweight; MUNW, metabolically unhealthy normal weight; MUO, metabolically unhealthy obesity; MUOW, metabolically unhealthy overweight; OR, odds ratio.

* *p* < 0.05.

** *p* < 0.01.

*** *p* < 0.001.

### 3.3 Correlation of different metabolic phenotypes with elevated UACR in subgroups

**FIGURE 2:**
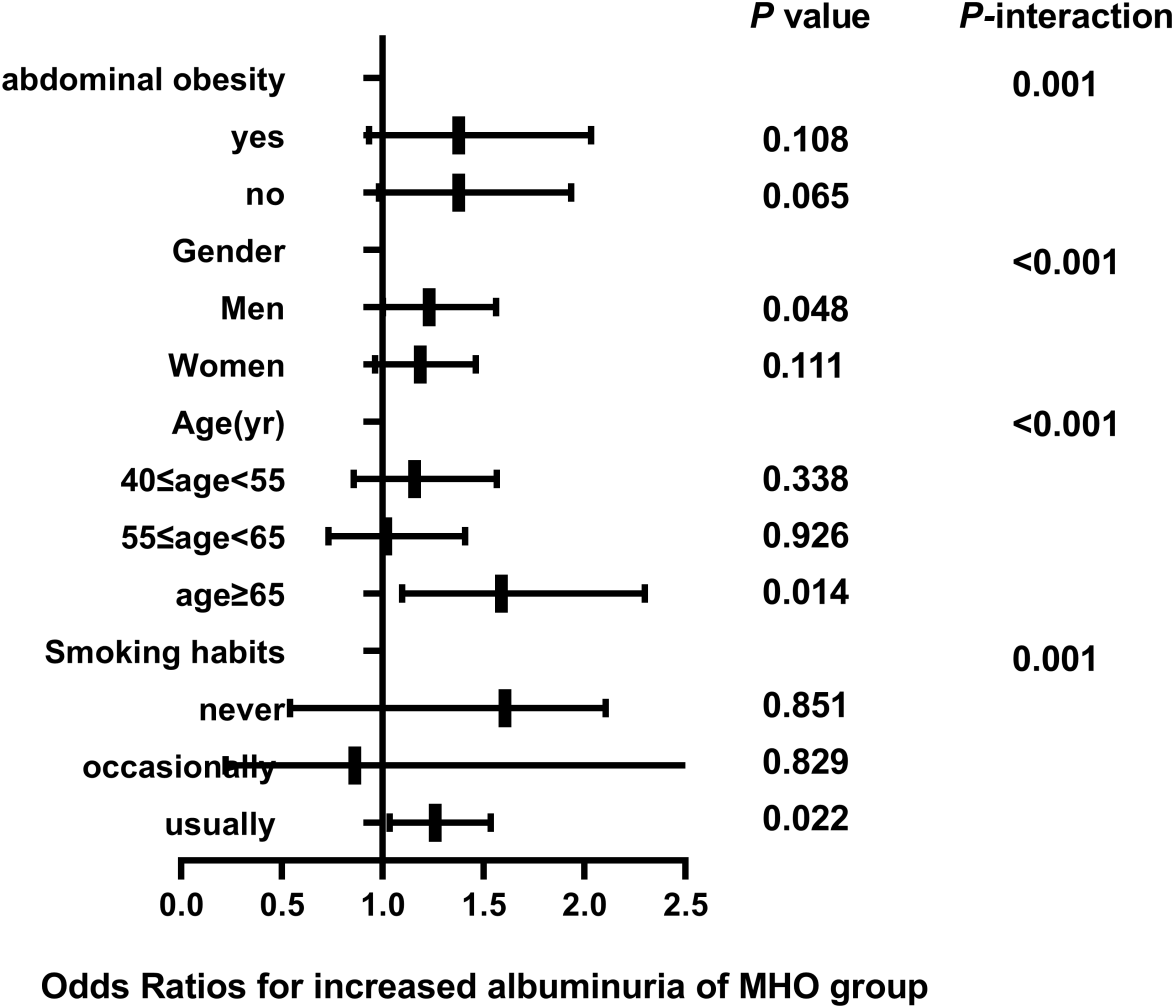
The risk of increased albuminuria in different subgroups of MHO group. All models are adjusted for age, centre, sex, education status, smoking habits, drinking habits, eGFR, LDL‐C, AST, GGT, HbA1c. MHO, metabolically healthy obesity

**FIGURE 3:**
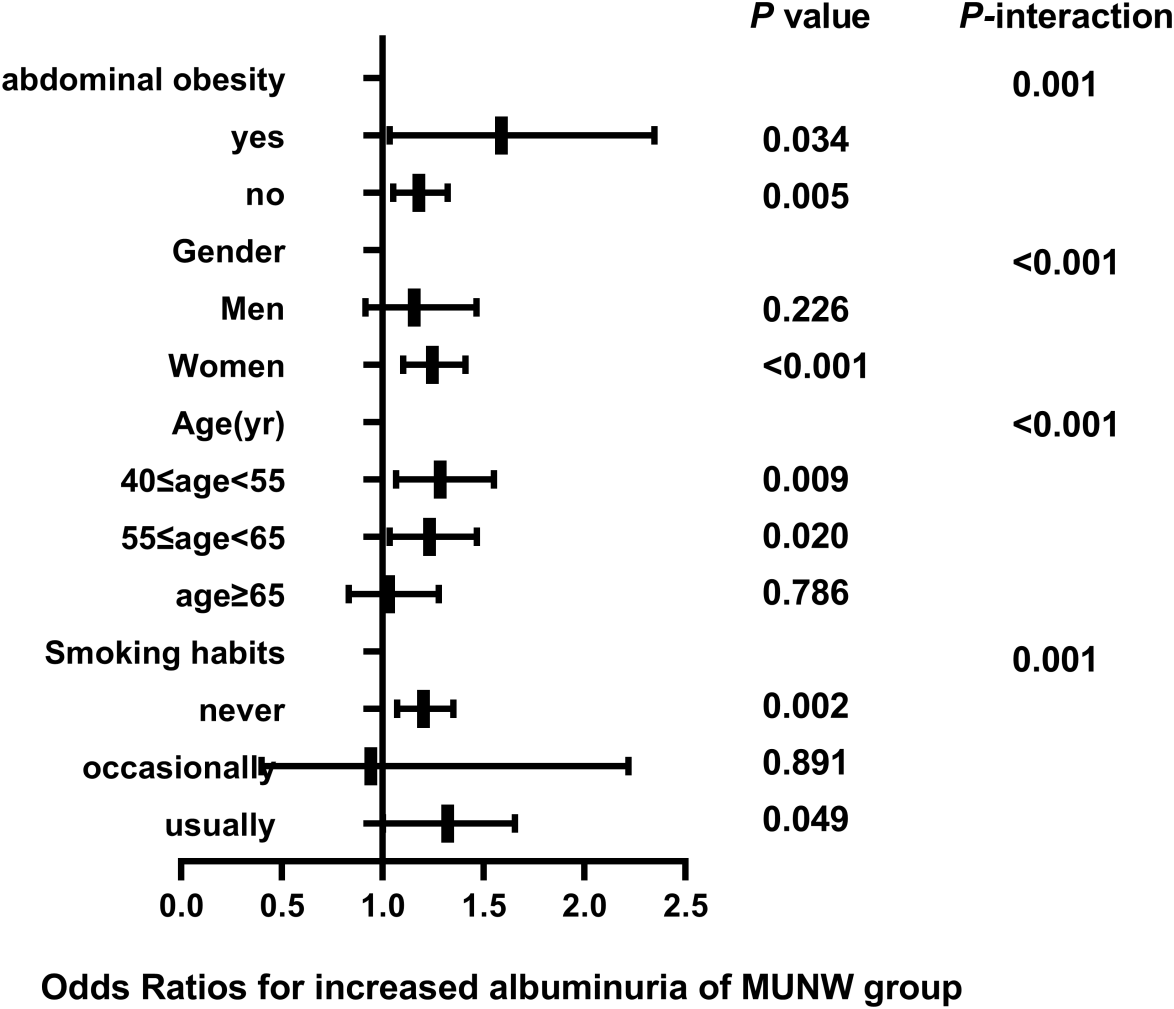
The risk of increased albuminuria in different subgroups of MUNW group. All models are adjusted for age, centre, sex, education status, smoking habits, drinking habits, eGFR, LDL‐C, AST, GGT, HbA1c. MUNW, metabolically unhealthy normal weight

**FIGURE 4:**
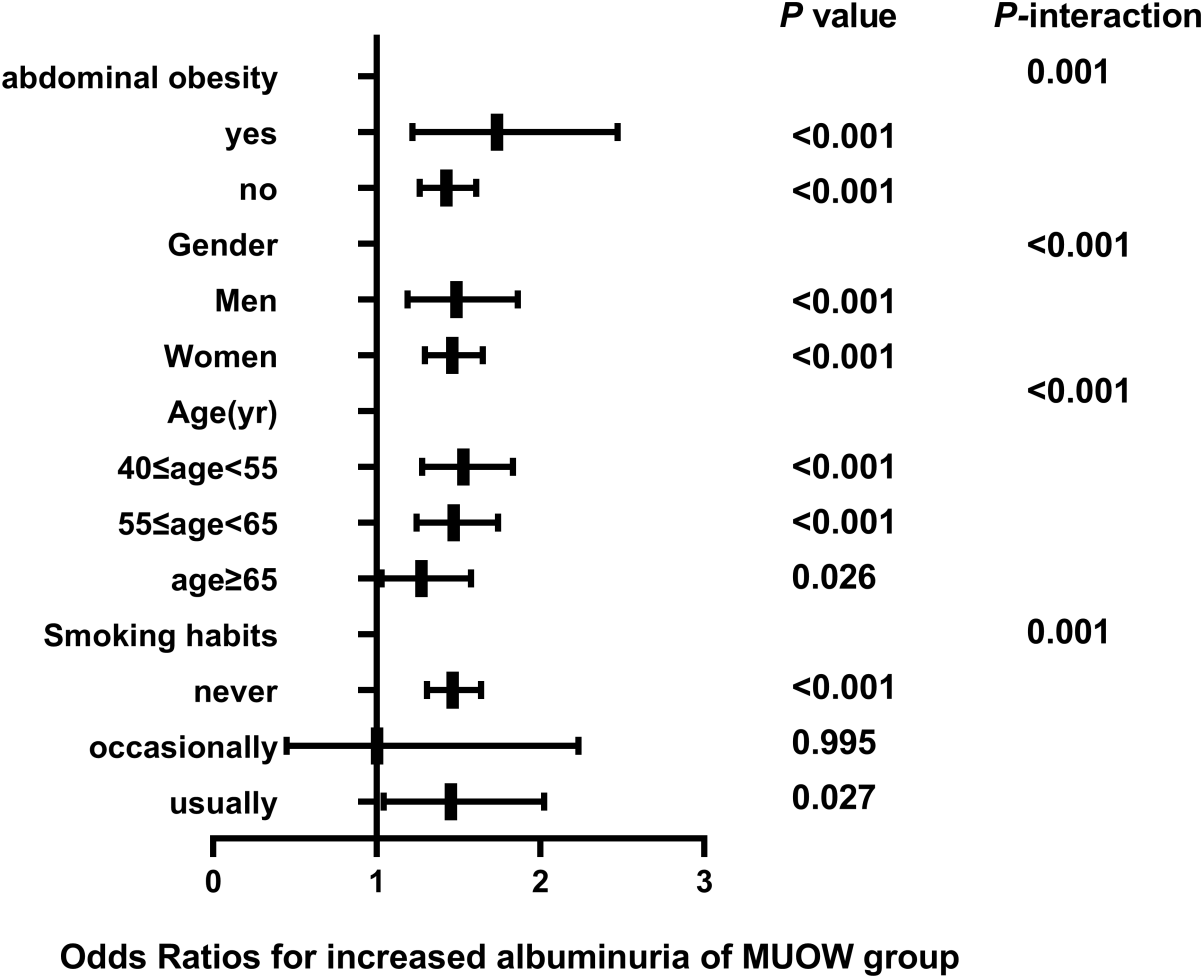
The risk of increased albuminuria in different subgroups of MUOW group. All models are adjusted for age, centre, sex, education status, smoking habits, drinking habits, eGFR, LDL‐C, AST, GGT, HbA1c. MUOW, metabolically unhealthy overweight

**FIGURE 5:**
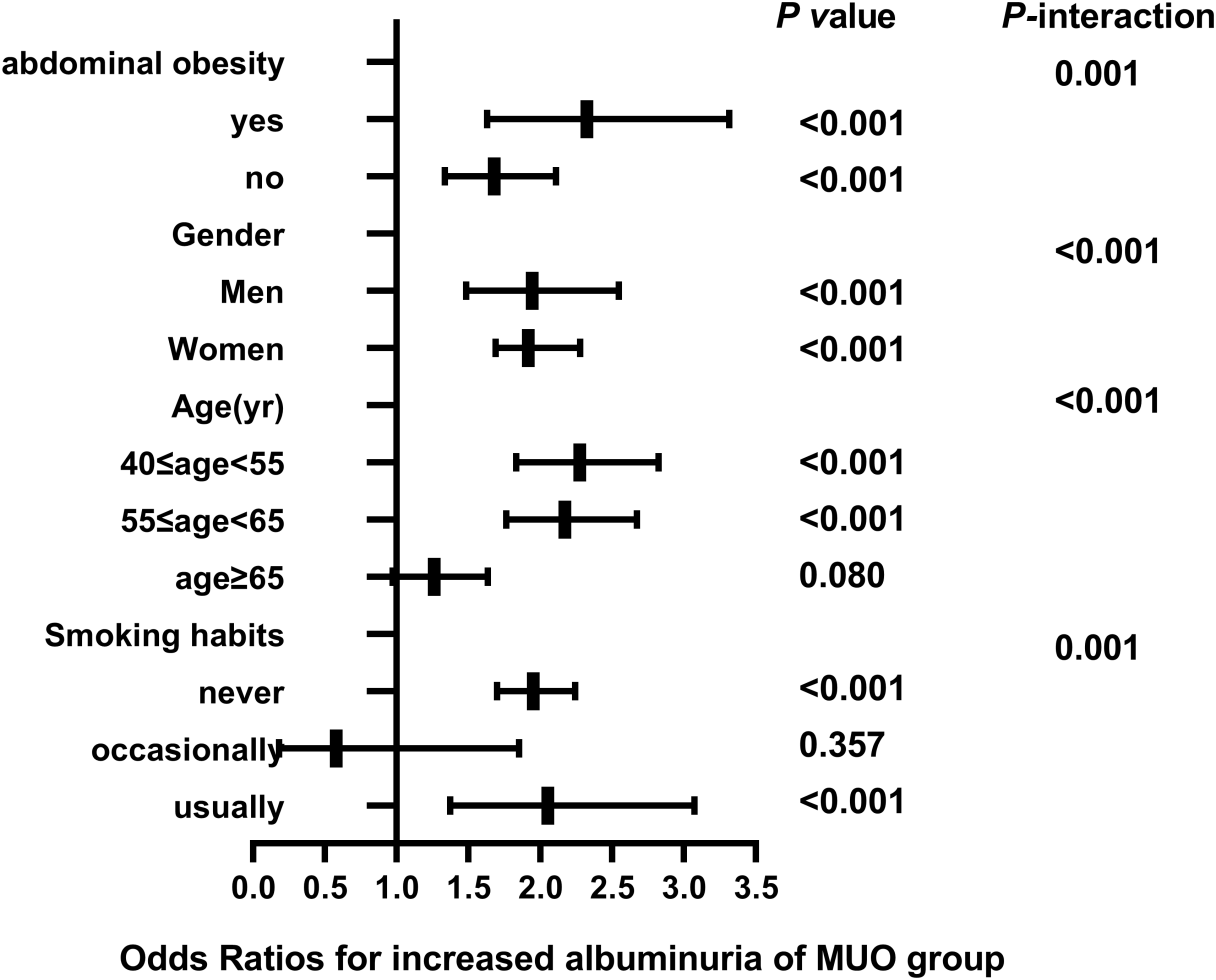
The risk of increased albuminuria in different subgroups of MUO group. All models are adjusted for age, centre, sex, education status, smoking habits, drinking habits, eGFR, LDL‐C, AST, GGT, HbA1c. MUO, metabolically unhealthy obesity

The correlation between different metabolic phenotypes and increased albuminuria in different populations was further explored, stratified by age (*P*_interaction_ for <.001), sex (*P*_interaction_ for <.001), abdominal obesity (*P*_interaction_ for .001), and smoking status (*P*_interaction_ for .001). The OR of the MHO group (Figure 2), MUNW group (Figure 3), MUOW group (Figure 4), and MUO group (Figure 5) were statistically significant. Stratifying the population by sex, the risk of increased albuminuria was greater in men than in women in the MUOW group (OR 1.488; 95% CI, 1.189–1.861; *p* < 0.001) and the MUO group (OR 1.945; 95% CI, 1.485–2.546; *p* < 0.001), using the MHNW group as the reference group. Stratifying the population by age showed the highest OR in the MUO group in all three age strata and the biggest risk of increased albuminuria in the MUO group among the three phenotypes of metabolic abnormalities (OR 2.275; 95% CI, 1.832–2.825; *p* < 0.001) in the 40–55 years group. In the smoking habit subgroup, the risk of increased albuminuria was elevated in the regular smokers of the MUOW group (OR 1.453; 95% CI, 1.043–2.023; *p* = 0.027) and MUO group (OR 2.053; 95% CI, 1.372–3.073; *p* < 0.001) compared to the nonsmoking population. Furthermore, we found that the incidence of proteinuria was highest in those with abdominal obesity, especially in the MUO group (OR 1.453, 95% CI, 1.043–2.023, *p* = 0.027). It is suggested that the risk of increased albuminuria is elevated among regular smokers in men aged 40–55 years in the population with abdominal obesity.

## 4 DISCUSSION

The main finding of this study was that the proportion of MHNW, MHOW, MHO, MUNW, MUOW, and MUO subjects in the Chinese community in the elderly population was 27.6%, 15.9%, 4.1%, 19.8%, 22.5%, and 9.6%, respectively, while the increased albuminuria in MHNW, MHOW, MHO, MUNW, MUOW, and MUO subjects was 8.6%, 8.9%, 11.4%, 12.6%, 14.1% and 16.9%, respectively. The risk of increased albuminuria was higher in MHO subjects than in MHNW subjects, and the risk of increased albuminuria was highest in MUO subjects; the risk of increased albuminuria elevates with increasing BMI.

A cross‐sectional study of 9579 Chinese middle‐aged and older adults found that low‐grade albuminuria was significantly associated with an increased incidence of MS and its components.^16^ The metabolic phenotype used in our study considered metabolic risk factors and BMI, which has been used to assess the risk of various outcomes such as CVD, CKD, etc. The prognostic value of MHO is a controversial topic, and it has been shown that compared to the MHNW group, the MHO group does not have an increased risk of cardiometabolic disorders and mortality,^17^ but in a large cohort study of Korean adults, the MHOW or MHO groups were associated with an increased risk of CKD compared to the MHNW group.^18^ Several cohort studies reported that the MHO group was not exempt from the development of type 2 diabetes mellitus events^19^ and CVD,^20^ suggesting that MHO is not a harmless condition. A meta‐analysis by Alizadeh et al^21^ analyzed nine prospective cohort studies comparing metabolic phenotype and risk of CKD. They found that the MHO group and MUNW group have an elevated risk of CKD development and a combined relative risk (RR) of 1.55 and 1.58, respectively. This is consistent with our findings of an elevated risk of increased albuminuria of 1.198 and 1.272 for the MHO phenotype and MUNW phenotype, respectively, suggesting that obesity is a risk factor for CKD regardless of the presence of metabolic abnormalities. Metabolic health does not fully protect the obese population.

It is also important to note that metabolic phenotypes are dynamic, with a high risk of transitioning from metabolically healthy phenotypes to metabolically unhealthy ones over time,^22^ as Soriguer et al^23^ found that 30%–40% of patients with MHO transition to the MUO status after 6 years of follow‐up. Therefore, appropriate interventions to improve MHO status and prevent its conversion to MUO may help to reduce the incidence of CKD.

Obesity is considered to be one of the strongest but most modifiable risk factors for renal insufficiency,^24^ and the association between obesity and increased albuminuria may be mediated by multiple biological mechanisms. First, obesity itself has independent deleterious effects on renal hemodynamics, including hyperfiltration, increased glomerular capillary wall tension, and podocyte stress^25^; and hyperfiltration is an independent predictor of albuminuria development.^26^ Second, adipose tissue, as an active endocrine organ, secretes adipose tissue‐derived adipokines^27^ and cytokines,^28^ such as leptin, lipocalin, tumor necrosis factor‐α, interleukin‐6, and fibrinogen activator inhibitor‐1, involved in the pathogenesis of CKD. Adipokines reach the kidney and exert local effects on thylakoid cells, podocytes, and tubular cells, promoting hyperfiltration of the glomerulus^29^ and albuminuria.^30^ Finally, albuminuria may also result from mechanisms such as systemic chronic low‐grade inflammation,^31^ increased insulin resistance,^32^ inappropriate activation of the renin‐angiotensin‐aldosterone system,^33^and increased oxidative stress.^34^

Metabolic abnormalities are also closely associated with increased albuminuria, with elevated blood pressure and increased insulin resistance being directly related to endothelial dysfunction and renal hemodynamic instability, leading to podocyte damage, which again leads to albuminuria.^35^ Obesity and hypertension can impair autoregulation of renal afferent small arteries, decrease podocyte density,^36^ and synergistically promote the development of albuminuria.^37^ In addition, an abnormal lipid profile can accelerate atherosclerosis through renal vascularity and fat deposition in the renal tubules, leading to endothelial cell inflammation and renal tubular interstitial injury.^38^Hyperglycemia leads to multiple metabolic disorders through activation of protein kinase C,^39^ resulting in renal dysfunction and glomerular hyperfiltration.

We chose to analyze the population stratified by age, sex, abdominal obesity, and smoking habits. First of all, age is a known independent risk factor for renal function impairment.^40^ Then, the protective effect of estrogen in women and/or the damaging effect of testosterone, combined with unhealthy lifestyles in men, may lead to a more rapid decline in renal function in men than in women.^41^ Finally, smoking is a well‐known risk factor for CKD.^42^ Our results suggest that men in the MUO group aged 40–55 years who are regular smokers and abdominally obese may have a higher risk of increased albuminuria. To reduce the risk of CKD, they should consider quitting smoking, losing weight, and making lifestyle changes, such as eating a balanced diet and exercising more.

The strength of this study is that it is the first study to analyze the relationship between different metabolic phenotypes and increased albuminuria in a large cross‐sectional sample; however, there are some limitations to consider in this study. First, the population of this study included only Chinese people aged 40 years or older, and the BMI grouping used in the study was based on the Working Group on Obesity in China (WGOC) obesity diagnostic criteria of the Chinese population study, so our findings may not apply to individuals of different ages or races. Second, due to the cross‐sectional nature of this study, the causal relationship between increased albuminuria and different metabolic phenotypes has not been established and needs to be further demonstrated in prospective studies. Third, we only tested serum albumin concentration in one urine sample. Multiple or 24‐h urine samples will provide more accurate and stable urinary albumin excretion data. Finally, we did not perform obesity typing, and it has been reported that the main reason for the different metabolic statuses of individuals with the same BMI is the different fat distribution patterns, with excess visceral fat being more detrimental to metabolic health than excess subcutaneous fat,^43^ which needs to be further explored.

## 5 CONCLUSION

Our study shows that there is a significant correlation between metabolic phenotype and increased albuminuria. In a community‐based Chinese elderly population, increased albuminuria is associated with increased BMI regardless of normal metabolism, and those with metabolic abnormalities are at greater risk of increased albuminuria than those with normal metabolism. The risk of increased albuminuria was further elevated among regular smokers among men aged 40–55 years with abdominal obesity. Since people who are overweight or obese have a higher risk of CKD, we recommend that they have their UACR checked regularly and develop an appropriate fat loss program to reduce their risk.

## DISCLOSURE

The authors declare that they have no conflicts of interest.

## References

[1] Adair KE , von Waaden N , Rafalski M , Hess BW , Weaver SP , Bowden RG . Metabolic phenotypes and chronic kidney disease: a cross‐sectional assessment of patients from a large federally qualified health center. Life (Basel). 2021;11(2):175. 33672432 10.3390/life11020175 PMC7926935

[2] Bikbov B , Purcell CA , Levey AS , Smith M . Global, regional, and national burden of chronic kidney disease, 1990–2017: a systematic analysis for the global burden of disease study 2017. Lancet. 2020;395(10225):709‐733. 32061315 10.1016/S0140-6736(20)30045-3 PMC7049905

[3] Smith ER , Cai MM , McMahon LP , Wright DA , Holt SG . The value of simultaneous measurements of urinary albumin and total protein in proteinuric patients. Nephrol Dial Transplant. 2012;27(4):1534‐1541. 22193048 10.1093/ndt/gfr708

[4] Ninomiya T , Perkovic V , de Galan BE , et al. Albuminuria and kidney function independently predict cardiovascular and renal outcomes in diabetes. J Am Soc Nephrol. 2009;20(8):1813‐1821. 19443635 10.1681/ASN.2008121270 PMC2723977

[5] Kelly T , Yang W , Chen CS , Reynolds K , He J . Global burden of obesity in 2005 and projections to 2030. Int J Obes. 2008;32(9):1431‐1437. 10.1038/ijo.2008.102 18607383

[6] Obermayr RP , Temml C , Knechtelsdorfer M , et al. Predictors of new‐onset decline in kidney function in a general middle‐european population. Nephrol Dial Transplant. 2008;23(4):1265‐1273. 18039642 10.1093/ndt/gfm790

[7] Association AM . Executive summary of the third report of the National Cholesterol Education Program (NCEP) expert panel on detection, evaluation, and treatment of high blood cholesterol in adults (adult treatment panel III). JAMA. 2001;285(19):2486‐2497. 11368702 10.1001/jama.285.19.2486

[8] Thomas G , Sehgal AR , Kashyap SR , Srinivas TR , Kirwan JP , Navaneethan SD . Metabolic syndrome and kidney disease: a systematic review and meta‐analysis. Clin J Am Soc Nephrol. 2011;6(10):2364‐2373. 21852664 10.2215/CJN.02180311 PMC3186450

[9] Popa S , Mota M , Popa A , Mota E , Serafinceanu C , Guja C . Prevalence of overweight/obesity, abdominal obesity and metabolic syndrome and atypical cardiometabolic phenotypes in the adult Romanian population: PREDATORR study. J Endocrinol Investig. 2016;39(9):1045‐1053. 27126310 10.1007/s40618-016-0470-4

[10] Wang Y , Zhu X , Chen Z , et al. Natural histories of metabolite BMI phenotypes and their impacts on cardiovascular disease risk over a decade‐long follow‐up. Obes Res Clin Pract. 2021;15(6):579‐586. 34742668 10.1016/j.orcp.2021.10.002

[11] Peng K , Chen G , Liu C , et al. Association between smoking and glycemic control in diabetic patients: results from the REACTION study. J Diabetes. 2017;10(5):408‐418. 10.1111/1753-0407.12625 29144059

[12] Stevens PE , Adeera Levin M , for the Kidney Disease: Members IGOCKDGDWG . Evaluation and Management of Chronic Kidney Disease: synopsis of the kidney disease: improving global outcomes 2012 clinical practice guideline. Ann Intern Med. 2013;158(11):825‐830. 23732715 10.7326/0003-4819-158-11-201306040-00007

[13] Cho YK , Lee J , Kim HS , et al. Impact of transition in metabolic health and obesity on the incident chronic kidney disease: a Nationwide cohort study. J Clin Endocrinol Metab. 2020;105(3):e148‐e157. 10.1210/clinem/dgaa033 31967306

[14] Pan X‐F , Wang L , Pan A . Epidemiology and determinants of obesity in China. Lancet Diabetes Endocrinol. 2021;9(6):373‐392. 34022156 10.1016/S2213-8587(21)00045-0

[15] Nishida C , Ko GT , Kumanyika S . Body fat distribution and noncommunicable diseases in populations: overview of the 2008 WHO expert consultation on waist circumference and waist‐hip ratio. Eur J Clin Nutr. 2010;64(1):2‐5. 19935820 10.1038/ejcn.2009.139

[16] Zhang J , Chen Y , Xu Y , et al. Low‐grade albuminuria is associated with metabolic syndrome and its components in middle‐aged and elderly Chinese population. PLoS One. 2013;8(6):e65597. 23805186 10.1371/journal.pone.0065597 PMC3689760

[17] Adair KE , Padgett RN , von Waaden N , Wilson RL , Bowden RG . Metabolic health, obesity, and cardiovascular disease: 2015‐2016 National Health and nutrition examination survey. Am J Med Sci. 2021;361(2):244‐252. 33531147 10.1016/j.amjms.2020.09.010

[18] Chang Y , Ryu S , Choi Y , et al. Metabolically healthy obesity and development of chronic kidney disease: a cohort study. Ann Intern Med. 2016;164(5):305‐312. 26857595 10.7326/M15-1323

[19] Bell JA , Kivimaki M , Hamer M . Metabolically healthy obesity and risk of incident type 2 diabetes: a meta‐analysis of prospective cohort studies. Obes Rev. 2014;15(6):504‐515. 24661566 10.1111/obr.12157 PMC4309497

[20] Aung K , Lorenzo C , Hinojosa MA , Haffner SM . Risk of developing diabetes and cardiovascular disease in metabolically unhealthy normal‐weight and metabolically healthy obese individuals. J Clin Endocrinol Metab. 2014;99(2):462‐468. 24257907 10.1210/jc.2013-2832 PMC3913817

[21] Alizadeh S , Esmaeili H , Alizadeh M , et al. Metabolic phenotypes of obese, overweight, and normal weight individuals and risk of chronic kidney disease: a systematic review and meta‐analysis. Arch Endocrinol Metab. 2019;63(4):427‐437. 31365625 10.20945/2359-3997000000149 PMC10528657

[22] Elias‐Lopez D , Vargas‐Vazquez A , Mehta R , et al. Natural course of metabolically healthy phenotype and risk of developing Cardiometabolic diseases: a three years follow‐up study. BMC Endocr Disord. 2021;21(1):85. 33910543 10.1186/s12902-021-00754-1 PMC8080399

[23] Soriguer F , Gutierrez‐Repiso C , Rubio‐Martin E , et al. Metabolically healthy but obese, a matter of time? Findings from the prospective Pizarra study. J Clin Endocrinol Metab. 2013;98(6):2318‐2325. 23559087 10.1210/jc.2012-4253

[24] Chang A , Kramer H . CKD progression: a risky business. Nephrol Dial Transplant. 2012;27(7):2607‐2609. 22555251 10.1093/ndt/gfs095

[25] Wickman C , Kramer H . Obesity and kidney disease: potential mechanisms. Semin Nephrol. 2013;33(1):14‐22. 23374890 10.1016/j.semnephrol.2012.12.006

[26] Kramer H , Reboussin D , Bertoni AG , et al. Obesity and albuminuria among adults with type 2 diabetes: the look AHEAD (action for health in diabetes) study. Diabetes Care. 2009;32(5):851‐853. 19196893 10.2337/dc08-2059 PMC2671132

[27] Garland JS . Elevated body mass index as a risk factor for chronic kidney disease: current perspectives. Diabetes Metab Syndr Obes. 2014;7:347‐355. 25114577 10.2147/DMSO.S46674 PMC4122576

[28] Hunley TE , Ma LJ , Kon V . Scope and mechanisms of obesity‐related renal disease. Curr Opin Nephrol Hypertens. 2010;19(3):227‐234. 20134323 10.1097/MNH.0b013e3283374c09 PMC2897176

[29] Helal I , Fick‐Brosnahan GM , Reed‐Gitomer B , Schrier RW . Glomerular hyperfiltration: definitions, mechanisms and clinical implications. Nat Rev Nephrol. 2012;8(5):293‐300. 22349487 10.1038/nrneph.2012.19

[30] D'Agati VD , Chagnac A , de Vries AP , et al. Obesity‐related glomerulopathy: clinical and pathologic characteristics and pathogenesis. Nat Rev Nephrol. 2016;12(8):453‐471. 27263398 10.1038/nrneph.2016.75

[31] Wahba IM , Mak RH . Obesity and obesity‐initiated metabolic syndrome: mechanistic links to chronic kidney disease. Clin J Am Soc Nephrol. 2007;2(3):550‐562. 17699463 10.2215/CJN.04071206

[32] Sarafidis PA , Ruilope LM . Insulin resistance, hyperinsulinemia, and renal injury: mechanisms and implications. Am J Nephrol. 2006;26(3):232‐244. 16733348 10.1159/000093632

[33] Ruster C , Wolf G . The role of the renin‐angiotensin‐aldosterone system in obesity‐related renal diseases. Semin Nephrol. 2013;33(1):44‐53. 23374893 10.1016/j.semnephrol.2012.12.002

[34] Ramos LF , Shintani A , Ikizler TA , Himmelfarb J . Oxidative stress and inflammation are associated with adiposity in moderate to severe CKD. J Am Soc Nephrol. 2008;19(3):593‐599. 18256365 10.1681/ASN.2007030355 PMC2391046

[35] Tsuda A , Ishimura E , Uedono H , et al. Association of Albuminuria with Intraglomerular Hydrostatic Pressure and Insulin Resistance in subjects with impaired fasting glucose and/or impaired glucose tolerance. Diabetes Care. 2018;41(11):2414‐2420. 30217931 10.2337/dc18-0718

[36] Fotheringham J , Kawar B , McKane W , Ellam T . Obesity modulates the association between systolic blood pressure and albuminuria. Nephrol Dial Transplant. 2018;33(4):607‐613. 29156004 10.1093/ndt/gfx081

[37] Chen HM , Liu ZH , Zeng CH , Li SJ , Wang QW , Li LS . Podocyte lesions in patients with obesity‐related glomerulopathy. Am J Kidney Dis. 2006;48(5):772‐779. 17059996 10.1053/j.ajkd.2006.07.025

[38] Lee SH , Kim DH , Kim YH , et al. Relationship between dyslipidemia and albuminuria in hypertensive adults: a Nationwide population‐based study. Medicine (Baltimore). 2016;95(16):e3224. 27100412 10.1097/MD.0000000000003224 PMC4845816

[39] Das Evcimen N , King GL . The role of protein kinase C activation and the vascular complications of diabetes. Pharmacol Res. 2007;55(6):498‐510. 17574431 10.1016/j.phrs.2007.04.016

[40] Retnakaran R , Cull CA , Thorne KI , Adler AI , Holman RR . Risk factors for renal dysfunction in type 2 diabetes. Diabetes. 2006;55(6):1832‐1839. 16731850 10.2337/db05-1620

[41] Carrero JJ , Hecking M , Chesnaye NC , Jager KJ . Sex and gender disparities in the epidemiology and outcomes of chronic kidney disease. Nat Rev Nephrol. 2018;14(3):151‐164. 29355169 10.1038/nrneph.2017.181

[42] Orth SR , Hallan SI . Smoking: a risk factor for progression of chronic kidney disease and for cardiovascular morbidity and mortality in renal patients—absence of evidence or evidence of absence? Clin J Am Soc Nephrol. 2008;3(1):226‐236. 18003763 10.2215/CJN.03740907

[43] De Lorenzo A , Soldati L , Sarlo F , Calvani M , Di Lorenzo N , Di Renzo L . New obesity classification criteria as a tool for bariatric surgery indication. World J Gastroenterol. 2016;22(2):681‐703. 26811617 10.3748/wjg.v22.i2.681 PMC4716069

